# Evolutionary origin of the *NCSI* gene subfamily encoding norcoclaurine synthase is associated with the biosynthesis of benzylisoquinoline alkaloids in plants

**DOI:** 10.1038/srep26323

**Published:** 2016-05-18

**Authors:** Sornkanok Vimolmangkang, Xianbao Deng, Albert Owiti, Thitirat Meelaph, Collins Ogutu, Yuepeng Han

**Affiliations:** 1 Key Laboratory of Plant Germplasm Enhancement and Specialty Agriculture, Wuhan Botanical Garden of the Chinese Academy of Sciences, Wuhan, 430074, P. R. China; 2 Department of Pharmacognosy and Pharmaceutical Botany, Faculty of Pharmaceutical Sciences, Chulalongkorn University, Bangkok 10330, Thailand; 3 Graduate University of Chinese Academy of Sciences, 19A Yuquanlu, Beijing, 100049, China; 4 Sino-African Joint Research Center, Chinese Academy of Sciences, Wuhan, 430074, China

## Abstract

Sacred lotus is rich in biologically active compounds, particularly benzylisoquinoline alkaloids (BIAs). Here, we report on isolation of genes encoding (*S*)-norcoclaurine synthase (NCS) in sacred lotus, which is a key entry-enzyme in BIA biosynthesis. Seven *NCS* genes, designated *NnNCS1* through *NnNCS7*, were identified in the sacred lotus genome, and five are located next to each other within a 83 kb region on scaffold 8. The *NCS* genes are divided into two subfamilies, designated *NCSI* and *NCSII*. The *NCSII* genes are universal in plants, while the *NCSI* genes are only identified in a limited number of dicotyledonous taxa that produce BIAs. In sacred lotus, only *NnNCS4* belongs to the *NCSII* subfamily, whilst the rest *NCS* genes within the *NCSI* subfamily. Overall, the *NnNCS7* gene was predominantly expressed in all tested tissues, and its expression is significantly correlated with alkaloid content in leaf. In contrast, the *NnNCS4* expression shows no significant correlation with alkaloid accumulation in leaf, and its lack of expression cannot inhibit alkaloid accumulation. Taken together, these results suggest that the *NCSI* subfamily is crucial for BIA biosynthesis, and its origin may represent an important evolutionary event that allows certain plant taxa to produce BIAs.

Sacred lotus (*Nelumbo nucifera* Gaertn.) is an ancient perennial aquatic plant wildly distributed in Eastern Asia. All parts of the lotus plant, including flowers, seeds, rhizomes, and leaves are edible, and some are also used as traditional Chinese herbal medicine^1^. For example, lotus leaves are used to treat dysentery, diarrhoea, dizziness, and blood vomiting, whereas, lotus flowers are used to treat symptoms such as pain, inflammation, bleeding due to internal and external injury, and skin disorders. Numerous studies have showed that lotus extract and isolated compounds have a variety of biological activities, such as anti-HIV^2^, anti-obesity^3^,^4^, antimicrobial^5^,^6^, anti-diabetic^7^, anti-platelet aggregation^8^, anti-cancer^9^,^10^,^11^,^12^, anti-acetylcholinesterase^13^,^14^, and a potential use in Huntington’s disease^15^.

Alkaloids are recognized as major bioactive constituents in sacred lotus^1^. The dominant type of alkaloids in sacred lotus is benzylisoquinoline alkaloids (BIAs), which are a structurally diverse group of compounds, including *N*-nornuciferine, *O*-nornuciferine, nuciferine, lotusine, nuferine, and roemerine, among others^16^. BIAs are present in all parts of sacred lotus, but their composition varies among tissue types^17^. In lotus cotyledons, neferine and lotusine are found, whereas in the leaves, nuciferine is dominant^18^.

BIAs are restricted to certain plant families, including Papaveraceae, Ranunculaceae, Berberidaceae, Menispermaceae, and Nelumbonaceae. To date, BIAs have been extensively investigated in pharmaceutical plants, such as opium poppy (*Papaver somniferum*), California poppy (*Eschscholzia californcia*), meadow rue (*Thalictrum* species), and Japanese goldthread (*Coptis japonica*), and approximately 2,500 BIAs have been reported^19^. BIAs include a range of chemicals used as drug treatments in humans, and the well-known BIA-derived drugs are strong analgesic morphine, antitussive codeine, antispasmodic papaverine, and antibiotic sanguinarine and berberine. Due to their huge diversity in plants, BIAs are divided into different classes, such as morphinan, protoberberine, and aporphine, according to the similarity of their carbon skeleton. In lotus, BIAs mostly are of aporphine type, but their biosynthesis remains largely unknown.

Although BIAs show a wide structural diversity, their biosynthetic pathway are all initiated at the first common committed step, where isoquinoline core structure is generated by norcoclurine synthase (NCS). NCS catalyzes condensation of dopamine and tyrosine-derived 4-hydroxylphenylacetaldehyde (4-HPAA) to form trihydroxyisoquinoline (s)-norcoclaurine, which is the central precursor of BIAs^20^. NCS was initially isolated and characterized in a meadow rue (*Thalictrum flavum* ssp. glaucum)^21^,^22^. Later, NCS activity in total soluble protein extracts were observed in various plant species including *N. nucifera*, showing variation between extracts from root, stem, and leaf 23. However, few studies have been reported on isolation and characterization of genes encoding NCS in plants.

The *NCS* gene is well known to have evolved from a PR10/Bet v 1 ancestor in certain plant taxa, and this evolutionary origin represents a crucial step leading to the biosynthesis of BIAs^22^,^23^. In this study, we report for the first time the isolation and characterization of the *NCS* gene family in sacred lotus. The *NCS* genes were found to be frequently duplicated in the sacred lotus genome, playing important role in alkaloid accumulation. Phylogenetic analysis showed that the *NCS* genes can be divided into two subfamilies, *NCSI* and *NCSII*. The former was identified in certain plant taxa producing BIAs, while the latter is present in various plant species either or not producing BIAs. This suggests that the origin of the *NCSI* gene may represent an important evolutionary event that allows certain plant taxa to produce BIAs. Our results will aid our understanding of the mechanisms underlying the accumulation of alkaloids, particularly BIAs in plants.

## Materials and Methods

### Plant materials

All sacred lotus accessions used in this study are maintained at Wuhan Botanical Garden of the Chinese Academy of Sciences (Wuhan, Hubei province, PRC). A total of 10 lotus accessions, Xiaojinluan (XJL), Simeihuang (SMH), WSL253, Lianxia (LX), Rongjiao (RJ), WSL40, Fenshiba (FSB), Xuehuou (XH), Yupeng (YP), and Shuimeiren (SMR), were selected for real time PCR analysis and quantification of alkaloid content. Leaf and petiole samples were collected at young and mature stages for all cultivars, whereas, petal samples were collected only at full bloom stage. All samples were immediately frozen in liquid nitrogen and stored in −75 °C freezer until use.

### Isolation of the *NCS* genes in sacred lotus

Seven pairs of primers (Table S1) were designed to amplify *NCS* genes using cDNA a template. The amplified cDNA fragments were inserted into pEASY-T1 vector (TransGen Biotech, Beijing, China) and subsequently sequenced. Protein sequence alignment was performed using web-based MUSCLE program (http://www.ebi.ac.uk/Tools/msa/muscle/) and prediction of signal peptide was carried out using SignalP4.1 (http://www.cbs.dtu.dk/services/), WoLF PSORT^24^, and Protcomp 9.0 (http://linux1.softberry.com/berry.phtml).

### Phylogenetic analysis

Amino acid sequences of the *NCS* genes were used for phylogenic analysis. Sequence alignment was performed using MUSCLE in MEGA6 program^25^, and adjusted manually, as necessary. The resulting data matrix was analyzed using equally weighted maximum parsimony (MP). Phylogenetic tree was constructed using Maximum likelihood method based on the JTT matrix-based model^26^. The topology with superior log likelihood value was selected. The bootstrap consensus tree was inferred from 1,000 replicates and the branches with less than 50% bootstrap replicates were collapsed.

### Gene expression profiling using quantitative real-time PCR (qRT-PCR)

Approximately 100 mg of each sample was finely ground in liquid nitrogen and then subjected to total RNA isolation using Universal Plant Total RNA Extraction Kit (BioTeke, Beijing, China) according to the manufacturer’s instructions. Removal of contaminated genomic DNA and cDNA synthesis were conducted in one step using TransScript® One-Step gDNA Removal and cDNA Synthesis SuperMix (TransGen Biotech, Beijing, China). Real-time PCR assay was performed in Applied Biosystems StepOnePlus^TM^ Real-Time PCR System (Applied Biosystems). The qRT-PCR assay was carried out in a total volume of 10 μL reaction mixture containing 5 μL of 2× SYBR Green I Master Mix (Takara, Dalian, China), 0.2 μM of each primer, and 100 ng of template cDNA. The amplification program consisted of 1 cycle of 95 °C for 10 min, followed by 40 cycles of 95 °C for 30 sec, and 60 °C for 30 sec. The accumulation of PCR products was continuously monitored. After the final qPCR cycle, melting curve analysis of the qPCR products was carried out by heating from 60 to 90 °C at a rate of 0.5 °C/sec. An actin gene, *GADPH* (GenBank accession no. XM_010244040), was used as a constitutive control^27^. The primer sequences are listed in Table S2. All analyses were performed in triplicates.

### Measurement of alkaloid content

Alkaloid extraction was conducted using an ultrasonic protocol as previously described^28^. Briefly, samples of different lotus tissues were ground into fine powder in liquid nitrogen with a mortar and pestle. Approximately 600 mg powder was macerated in 7 mL of extraction buffer (0.3 M HCl-methanol, 1:1 in volume), and sonicated for 30 min at room temperature. After centrifugation (11,000 g, 10 min, room temperature), the supernatant was transferred to a new 15 mL eppendorf tube, and the pellet was extracted once again with 5 mL of extraction buffer. The supernatants were combined, and the final volume was adjusted to 15 ml with the extraction buffer.

Alkaloid extracts were filtered through 0.22 μm membranes (Shanghai New Asia purification device factory, Shanghai, China) and then analyzed using high-performance liquid chromatography (HPLC) with the same facility and method as we previously reported^29^. Total alkaloid concentration was determined with a standard curve (Fig. S1) designed with a commercial Nuciferine standard (Yuanye Biotechnology, Shanghai, China). For all tested tissues, three biological samples were measured in this study.

## Results

### Genes encoding NCS in the sacred lotus genome

We checked the annotation of the sacred lotus genome^30^, and four putative *NCS* genes, designated *NnNCS1* through *NnNCS4*, were identified, with accession numbers of NNU_21731-RA, NNU_21732-RA, NNU_14334-RA, and NNU_21730-RA, respectively, in the public domain (lotus-db.wbgcas.cn). To validate their putative coding sequences, primer pairs for each *NCS* gene were designed to amplify cDNAs prepared from young leaf of cv. WSL253 and the PCR products were subsequently cloned and sequenced. As a result, full coding sequences of three *NCS* genes, *NnNCS1*, *NnNCS3*, and *NnNCS4*, were successfully isolated and deposited in GenBank with accession numbers KT963033, KT963034, and KT963035, respectively. Meanwhile, several attempts were made to isolate cDNA fragments of *NnNCS2* from three different tissues (leaf, petiole, and petal) of four cultivars, Lianxia, WSL253, Fenshiba, and Shuimeiren. However, no PCR products were amplified. We checked the genomic DNA sequence of the putative *NnNCS2* gene, and found that it showed a high level (95%) of similarity with *NnNCS1*. However, *NnNCS2* had a single nucleotide deletion adjacent to the GC dinucleotide at the 5′ border of the intron (Fig. S2). This single nucleotide deletion caused frameshift, resulting in a truncated and nonfunctional protein due to a premature stop codon. Thus, *NnNCS2* is deemed to be a pseudogene.

**Figure 1:**
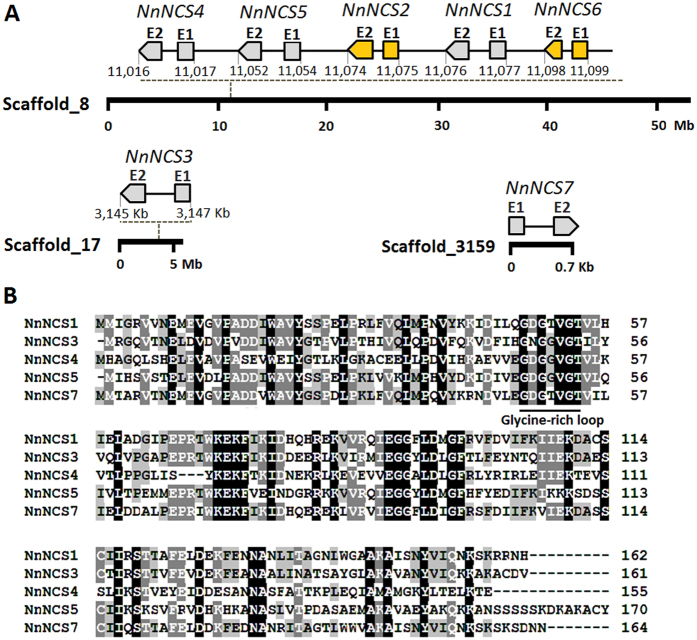
Genes encoding norcoclaurine synthase in sacred lotus. (**A**) Physical position and genomic structure of *NnNCS*s in the lotus genome. The pseudogenes are highlighted in yellow background. (**B**) Alignment of deduced amino acid sequences of five *NnNCS* genes in sacred lotus. The conserved glycine-rich loop represents the characteristic of Bet v1 family proteins.

To determine the copy gene number of *NCS* in lotus, the coding sequences of *NnNCS1* were compared against the draft genome of sacred lotus ‘China Antique’^30^. As a result, three additional *NCS* paralogs, termed *NnNCS5, NnNCS6*, and *NnNCS7*, were identified. The full coding sequences of *NnNCS5* were confirmed by whole cDNA amplification and sequencing. *NnNCS7* was initially identified to be located on scaffold_3159, but this scaffold is very short and only covers the first exon. The second exon was subsequently recovered by PCR-based genome working. The cDNA sequences of *NnNCS5* and *NnNCS7* were deposited in GenBank with accession numbers KU234431 and KU234432, respectively. However, the putative *NnNCS6* gene contained only partial coding sequences (Fig. S3), and its transcripts were not detected in three different tissues (leaf, petiole, and petal) of four cultivars, Lianxia, WSL253, Fenshiba, and Shuimeiren. Thus, *NnNCS6* is deemed to be a pseudogene. In addition, *NnNCS3* was located on scaffold 17, while *NnNCS1*, *NnNCS2*, *NnNCS4, NnNCS5*, and *NnNCS6* were located next to each other within an 83 kb region on scaffold 8 (Fig. 1A).

All the *NnNCS* genes were composed of two exons and one intron. The coding-sequence lengths of *NnNCS1*, *NnNCS3*, *NnNCS4*, *NnNCS5* and *NnNCS7* were 489, 486, 468, 513, and 492 bp, respectively. *NnNCS1* and *NnNCS7* shared the highest level (83.8%) of identity in coding DNA sequences, and they showed approximately 65% identity in coding DNA sequences with *NnNCS3* and *NnNCS5*. *NnNCS4* had approximately 55% identity in coding DNA sequences with other *NnNCS* genes. The deduced amino acid sequences of *NnNCS1*, *NnNCS3*, *NnNCS4*, *NnNCS5*, and *NnNCS7* genes were 162, 161, 155, 170, and 164 in length, respectively. *NnNCS1*, *NnNCS3*, *NnNCS5* and *NnNCS7* genes shared over 50% identity in amino acid sequences with each other, while they showed approximately 40% identity in amino acid sequences with *NnNCS4*. In addition, all the deduced amino acid sequences of the *NnNCS* genes contained a glycine-rich loop (Fig. 1B), the ligand binding domain of Bet v1 protein family. Subcellular localization of the NnNCS amino acid sequences was predicted using several programs, including SignalP4.1, WoLF PSORT, and Protcomp 9.0. However, the result indicated that none of the lotus NnNCS proteins contained any signal peptides. This suggests that the lotus NnNCS proteins are probably localized in cytoplasm.

### Phylogenetic analysis of *NCS* genes and their homologs

The NCS enzyme belongs to Bet v 1 protein family and its activity was biochemically characterized in a limited number of species, including opium poppy (*Papaver somniferrum*)^23^, yellow meadow-rue (*Thalictrum flavum*)^21^,^31^, Japanese goldthread (*Coptis japonica*)^32^, and *Sinopodophyllum hexandrum*^33^. Besides NCS, Bet v 1 family also includes plant intracellular pathogenesis-related class 10 (PR-10) proteins, cytokinin binding proteins (CSBPs), major latex proteins (MLPs), and ripening-related proteins. However, the PR-10/Bet v1 allergen proteins do not possess NCS activity although they share similarity to NCS proteins^34^. Thus, phylogenetic analysis was conducted to investigate the relationship between the lotus *NnNCS* genes and members of the Bet v1 family.

**Figure 2:**
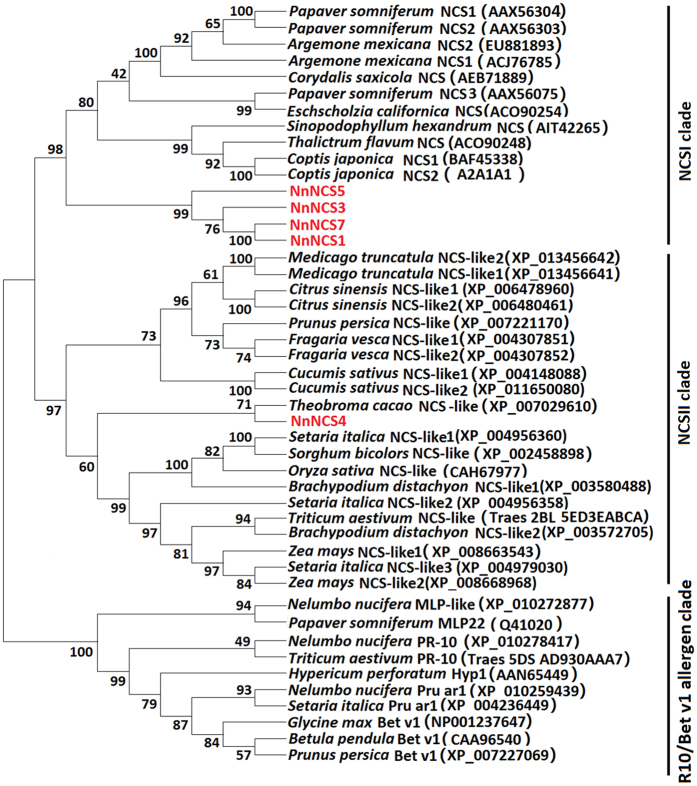
Phylogenetic relationship of *NCS* genes and their related homologs in plants. The amino acid sequences were retrieved from GenBank and Phytozome database, and their accession numbers are indicated in brackets. MLP, PR-10, Pru ar1, Hyp1, Bet v1 represent the members of Bet v1 protein family that lack of NCS function. Genes isolated in this study are highlighted in red color.

All the *NCS* genes tested were divided into two subfamilies, designated *NCSI* and *NCSII* clades, but separated from the PR-10/Bet v1 allergen family (Fig. 2). The *NCSI* subfamily consisted of four *NnNCS* genes, *NnNCS1*, *NnNCS3*, *NnNCS5*, and *NnNCS7*, and functionally characterized *NCS* genes from Ranunculus such as California poppy, opium poppy, Japanese goldthread, and yellow meadow-rue. The *NCSII* subfamily was composed of the lotus *NnNCS4* gene and *NCS* genes from both eudicots, such as tea, cotton, and cacao, and monocots, such as rice, wheat, and maize.

### Gene expression profiles of the *NnNCS* genes in sacred lotus

**Figure 3:**
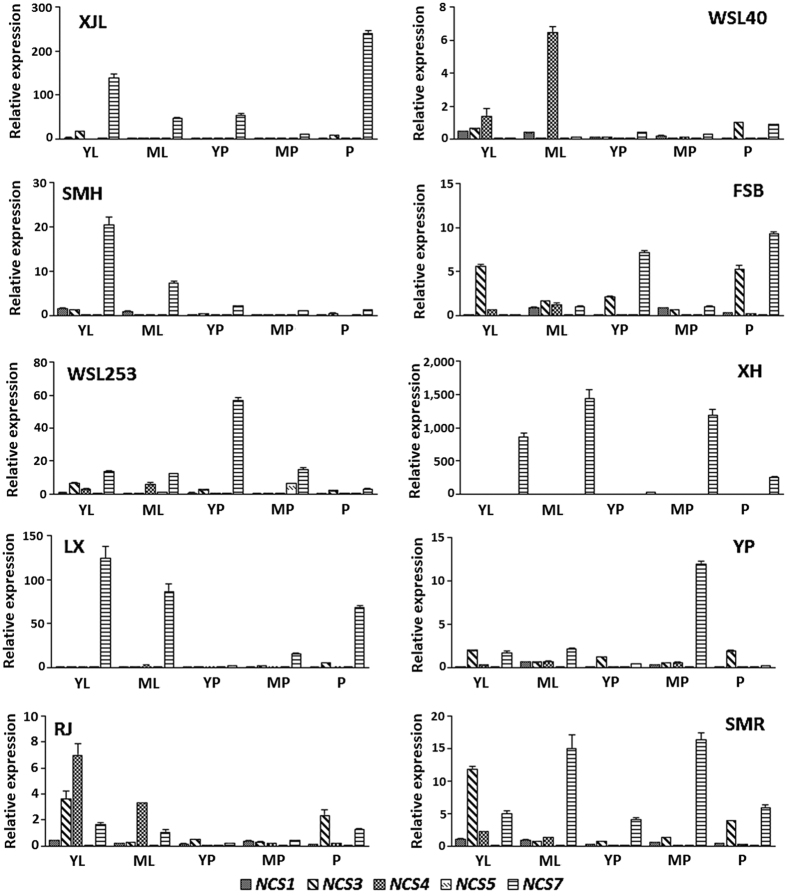
*NCS* gene expression profiles in flower-producing cultivars of lotus. YL, young leaf; ML, mature leaf; YP, young petiole; MP, mature petiole; and P, petal.

**Figure 4:**
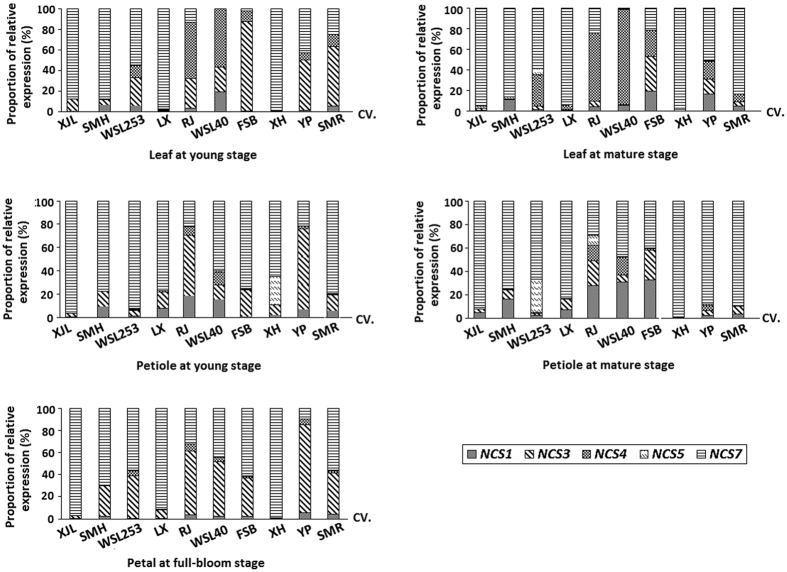
The proportion of different *NnNCS* transcripts in various tissues of fifteen lotus cultivars.

Spatial and temporal expression patterns of the five lotus *NCS* genes, *NnNCS1*, *NnNCS3*, *NnNCS4*, *NnNCS5*, and *NnNCS7*, were investigated in 10 cultivars (Fig. 3). Overall, *NnNCS7* was the predominant *NnNCS* transcript in leaf, petiole, and petal of most tested cultivars (Fig. 4). In contrast, the transcript levels of *NnNCS5* were extremely low or undetectable in leaf, petiole, and petal of most tested cultivars, but *NnNCS5* was relatively highly expressed in leaf and petiole of cv. Xuehuou. Similarly, *NnNCS4* showed extremely low or undetectable expression levels in leaf, petiole, and petal of most tested cultivars, but predominantly expressed in young and mature leaves of two cultivars, Rongjiao and WSL40. The transcripts of the remaining two *NnNCS* genes, *NnNCS1* and *NnNCS3*, were detected in all tested tissues. Expression of *NnNCS3* was high in petioles of three cultivars, Rongjiao, Fenshiba, and Yupeng, and in petals of most tested cultivars, such as Simeihuang, WSL253, Rongjiao, WSL40, Fenshiba, Yupeng and Shuimeiren. However, the expression levels of *NnNCS1* were very low compared with *NnNCS7* and/or *NnNCS3* transcripts. In addition, the tested cultivars were composed of white-flower and pink-flower genotypes. However, no relationship was observed between the expression pattern of *NnNCS* genes and the flower color.

### Total alkaloid content in various tissues of sacred lotus

Total alkaloid content was measured for the same accessions selected for qRT-PCR analysis (Table 1). On average, the alkaloid contents in young leaf, mature leaf, young petiole, mature petiole, and petal were 2.28, 2.26, 0.34, 0.19, and 0.35 mg/g FW, respectively. There was a wide range of alkaloid content in leaf across the tested cultivars, while a relatively narrow range of alkaloid content was observed for either petiole or petal across the tested cultivars. Overall, alkaloids were most abundant in the leaf, with a 6 to 12-fold higher amount as compared to both petiole and petal. The alkaloid content in the leaf ranged from 0.66 to 4.32 mg/g FW. Three cultivars, Lianxia, Rongjiao, and Simeihuang, had higher levels of alkaloids in young leaf than in mature leaf, whilst three cultivars, Xiaojinluan, Yupeng, and Fenshiba, showed higher levels of alkaloids in mature leaf than in young leaf. The remaining cultivars, WSL253, Xuehuou, Shuimeiren, and WSL40, showed no great difference in total alkaloid content between young and mature leaves.

Alkaloid content in petiole ranged from 0.08 to 0.50 mg/g FW. Five cultivars, Rongjiao, Xiaojinluan, Simeihuang, Yupeng, and Fenshiba, showed 2- to 3-fold decrease in total alkaloid content compared with young petiole, while a slight decrease was observed for the rest five cultivars. On average, mature petiole showed an approximately 2-fold decrease in total alkaloid content compared with young petiole. In petal, the highest level of alkaloid accumulation (0.88 mg/g FW) was observed for cv. Yupeng bearing red-colored flowers, whilst the lowest level of alkaloid accumulation (0.07 mg/g FW) was observed for white flower cultivar Xiaojinluan and red flower cultivar Fenshiba. No relationship was observed between alkaloid accumulation and petal color.

### Relationship between the *NnNCS* gene expression and alkaloid accumulation

To investigate the potential role of *NCS* genes in alkaloid accumulation, we analyzed the relationship between the *NCS* gene expression and alkaloid accumulation. *NnNCS4* showed almost undetectable expression levels in leaf, petiole, and petal of three cultivars, Xiaojinluan, Simeihuang, and Xuehuou. Similarly, *NnNCS5* showed almost undetectable levels of expression in leaf, petiole, and petal of four cultivars, Shuimeiren, WSL40, Yupeng, and Fenshiba. As mentioned above, all tested cultivars accumulated alkaloids in different tissues, with relative abundance of alkaloids in young and/or mature leaves. These results indicated that both *NnNCS4* and *NnNCS5* play little role in alkaloid accumulation in sacred lotus.

The other three *NCSI* genes, *NnNCS1*, *NnNCS3*, and *NnNCS7*, were expressed in various tissues of all tested cultivars. Since bisbenzylisoquinoline-type alkaloids are synthesized mainly in lamina^29^, we further conducted correlation analysis to investigate the degree of linear relationship between the *NCS* gene expression level and alkaloid content in leaf. The expression of *NnNCS7* (*r* = −0.404, *P* < 0.05) was significantly correlated with alkaloid contents in leaf, whereas, no significant correlation was identified for either *NnNCS1* (*r* = 0.140, *P* > 0.05) and *NnNCS3* (*r* = 0.110, *P* > 0.05).

Taken together, the results above suggested that the *NCSI* genes are involved in the biosynthesis of alkaloids in sacred lotus.

## Discussions

Secondary metabolites play a crucial role in the adaptation of plants to their environment and represent an important source of pharmaceuticals. Plant secondary metabolites are highly diversified, which is obviously related to recruitment of genes for secondary metabolism. Gene duplication is assumed to be a major driving force for evolution of genes involved in secondary metabolism, and duplicated genes are often in tandem repeats, forming clusters within the plant genome^35^,^36^. For example, tandem duplication has been frequently observed for genes encoding enzymes involved in flavonoid biosynthesis, such as chalcone isomerase in soybean^37^ and *Lotus japonicus*^38^ and flavonoid 3-*O*-glycosyltransferase in peach^39^ and grapevine^40^. Two clusters of genes *O*-methyltransferases involved in lignin biosynthesis have been identified in the apple genome^41^. A cluster of three genes encoding terpene synthases involved in volatile biosynthesis have been identified in *Arabidopsis*^42^ and tomato^43^. More recently, tandem duplication of genes encoding known enzymes of the caffeine biosynthetic pathway has been proven to be crucial for the convergent evolution of caffeine biosynthesis in coffee^44^.

In this study, a cluster of five *NnNCS* genes involved in alkaloid biosynthesis was identified on linkage group 8 of sacred lotus. Among these clustered genes, *NnNCS1* showed high levels of nucleotide sequence identity with *NnNCS2* and *NnNCS6*. This suggests that *NnNCS1*, *NnNCS2*, and *NnNCS6* share a common progenitor, but *NnNCS2* and *NnNCS6* become pseudogenes due to the accumulation of deletions and/or point mutations. Besides the *NnNCS* cluster, two additional *NnNCS* genes, *NnNCS3* and *NnNCS7*, were also identified in the sacred lotus genome, but they have not been physically mapped onto any linkage groups. *NnNCS3* and *NnNCS7* are phylogenetically closely related to *NnNCS1*. The sacred lotus genome lacks the paleo-triplication observed in other eudicots^30^. Thus, it is reasonable to speculate that the *NnNCS1*, *NnNCS3*, and *NnNCS7* genes could have arisen by tandem duplication from a common progenitor. In addition, we checked the assembled sequence of the *NnNCS* cluster, and several unsequenced gaps remain in the gene cluster^30^. Further studies are needed to clarify whether *NnNCS3* and *NnNCS7* are also located within the *NnNCS* cluster on linkage group 8.

NCS is an important enzyme catalyzing the first committed step in benzylisoquinoline alkaloid biosynthesis in plants. In this study, we retrieved the *NCS* gene sequences from the genome sequences of eleven plant species, including dicots, such as sweet orange, strawberry, peach, *Medicago truncatula*, cucumber, and cacao, and monocots, such as maize, rice, *Brachypodium distachyon*, sorghum, and millet. These *NCS* genes along with previously characterized *NCS* genes were subjected to phylogenetic analysis, and the result indicates that all the *NCS* genes are divided into two subfamilies, *NCSI* and *NCSII*. Overall, the *NCSII* genes are present in all tested species, including both monocots and dicots, while the *NCSI* genes are identified in a limited number of dicotyledonous taxa that produce benzylisoquinoline alkaloids. A possible explanation for this finding is that the *NCSI* genes likely evolved through duplication of the *NCSII* genes in certain plant taxa. This explanation appears to be true in sacred lotus, in which the *NCSII* gene *NnNCS4* is clustered with the *NCSI* genes, such as *NnNCS1* and *NnNCS5*, and it has a closer relationship with the *NCSII* genes in monocots than those in dicots. Therefore, the *NnNCSI* genes in sacred lotus are likely derived fromduplication of the *NnNCSII* ancestor.

An ancient recruitment of the *NCS* gene from a PR10/Bet v 1 ancestor is proposed to be the primary evolutionary event that allowed certain plant taxa to produce BIAs^22^,^23^. As mentioned above, the *NCSI* gene probably evolved from the *NCSII* ancestor. Thus, it seems that the *NCSII* gene was initially recruited from a PR10/Bet v 1 ancestor, and its subsequent duplication resulted in the generation of *NCSI* genes. The *NCSII* gene is not only present in BIA-producing plant species, but also in plant species devoid of BIAs, such as Poaceae and Rosaceae^23^,^45^. In contrast, the *NCSI* gene is exclusively present in certain plant taxa that produce BIAs. These results suggest that evolutionary origin of *NCSI* genes from the *NCSII* ancestor could be associated with BIA biosynthesis in plants. In addition, previous study raised a question whether *Arabidopsis* contain the *NCS* gene^46^. We compared the amino acid sequences of the sacred lotus *NnNCS* genes against the *Arabidopsis* genome sequences using the BLASTP program, and only two Bet v 1 homologs were identified. This suggests that the *Arabidopsis* genome lacks any *NCS* genes, which is consistent with its deficiency in BIAs. Given gene loss has been frequently observed in *Arabidopsis*^47^, the *NCSII* gene is thus deemed to be lost during its evolution.

NCS plays an important role in the regulation of BIA biosynthesis due to its entry-point location in the pathway^48^. Our study indicates that the leaves of sacred lotus contain abundant BIAs, which is consistent with the previous report^1^,^16^. The expression level of the *NCSII* gene *NnNCS4* (*r* = 0.278, *P* > 0.05) shows no significant correlation with alkaloid content in leaves, and lack of the *NCSII* expression does not inhibit alkaloid accumulation. In contrast, *NnNCS7* are highly expressed in leaves and shows a significant correlation with alkaloid content. It is worth noting that the correlation is negative, which suggests that high or low levels of alkaloids may inhibit or induce, respectively, the expression of *NnNCS7* via feedback mechanism. Moreover, it is also worth noting that BIAs occur in Rutaceae^49^,^50^. *Citrus* plants belong to the family Rutaceae, and they produce alkaloids, such as betaine, citracridone, octopamine, synephrine, tyramine, and *N*-methyltyramine, but with no BIAs^51^,^52^. As mentioned above, sweet orange (*Citrus sinensis*) contains two copies of *NCSII* genes, but lacks any *NCSI* genes. These results further demonstrate that the *NCSI* genes, rather than the *NCSII* genes, are crucial for BIA biosynthesis in plants. To date, all the functional *NCS* genes previously characterized belong to the *NCSI* subfamily, while no members of the *NCSII* subfamily have been functionally characterized. It is worthy of further study to ascertain that the *NCSII* gene has no NCS activity. In addition, previous study suggests that the NCS proteins appear to assemble as dimers for their catalytic activities^21^. Besides *NnNCS7*, other *NCSI* genes such as *NnNCS1* and *NnNCS3* are also expressed in leaves of sacred lotus. More studies are still needed to clarify whether *NnNCS7* can act as a heterodimer with other *NCSI* genes to induce the biosynthesis of BIAs.

In general, our study shows that the sacred lotus leaves have the most abundant *NCS* transcripts, followed by petiole and petal. This is consistent with the finding that bisbenzylisoquinoline-type alkaloids are synthesized mainly in lamina^29^. Unlike sacred lotus, *Thalictrum flavum* ssp. glaucum and *Papaver somniferum* have low level of the *NCS* transcripts in leaf, but the *NCS* transcripts are abundant in rhizome/petiole^22^ and root/stem^34^, respectively. Overall, these studies show that the *NCS* gene expression is correlated with alkaloid accumulation in different tissues. The sacred lotus petiole predominantly accumulates the transcripts of the *NCSI* gene, *NnNCS7*, and its transcript level is significantly correlated with alkaloid accumulation. BIAs accumulate in the cytoplasm of specialized cells, also known as laticifers^49^,^53^. The sacred lotus leaf and petiole contain laticifers, in which latex sap is produced^54^. These results suggest that petiole is likely able to produce BIAs. However, a previous study showed that NCS activity was only observed in leaf, but not in petiole (stem) and root of lotus^23^. Here, *NnNCS7* is also highly expressed in petiole of sacred lotus, however with no significant correlation (*r* = −0.332, *P* > 0.05) with alkaloid content in petiole. Thus, further studies are still needed to address whether NnNCS7 plays similar roles in leaf and petiole.

Currently, lotus leaf has attracted more and more interest because of its use in tea/beverage production. Alkaloids are one of the most active ingredients in lotus leaf 55. Thus, understanding the mechanism underlying natural variation in alkaloid content will be useful for development of new rhizome-producing lotus cultivars with high level of alkaloid accumulation.

## Additional Information

**How to cite this article**: Vimolmangkang, S. *et al.* Evolutionary origin of the *NCSI* gene subfamily encoding norcoclaurine synthase is associated with the biosynthesis of benzylisoquinoline alkaloids in plants. *Sci. Rep.* **6**, 26323; doi: 10.1038/srep26323 (2016).

## Supplementary Material

Supplementary Information

## Figures and Tables

**Table 1:** Total alkaloid contents in lotus tissues (mg/g FW).

Cultivar	Petal color	Leaf		Petiole		Petal
		Young	Mature	Young	Mature	
Lianxia	White	4.32	2.69	0.33	0.28	0.68
WSL253	White	1.96	2.55	0.34	0.30	0.22
Rongjiao	White	3.31	1.70	0.48	0.17	0.64
Xiaojinluan	White	2.19	3.29	0.50	0.24	0.07
Simeihuang	White	2.56	1.58	0.25	0.13	0.12
Shuimeiren	Pale pink	3.77	2.82	0.37	0.20	0.12
Xuehuou	Pink	0.91	0.87	0.11	0.10	0.58
WSL40	Red	2.32	2.39	0.30	0.24	0.11
Yupeng	Red	0.84	1.48	0.40	0.12	0.88
Fenshiba	Red	0.66	3.22	0.28	0.08	0.07
Average		2.28	2.26	0.34	0.19	0.35

Top three highest amounts in each tissue were highlighted in bold. N/A, not applicable.
