# Supplementary material for: Evolutionary origin of the NCSI gene subfamily encoding norcoclaurine synthase is associated with the biosynthesis of benzylisoquinoline alkaloids in plants
Source: Sci Rep. 2016 May 18;6:26323. doi: 10.1038/srep26323 (PMC4870700; doi:10.1038/srep26323)
Supplement: Supplementary Information [file srep26323-s1.doc]

**Supporting Information for**

**Evolutionary origin of the *NCSI* gene subfamily encoding norcoclaurine synthase is associated with the biosynthesis of benzylisoquinoline alkaloids in plants**

Sornkanok Vimolmangkang^1,2†^, Xianbao Deng^1†^, Albert Owiti^1,3^, Thitirat Meelaph^2^, Collins Ogutu^1,3^, Yuepeng Han^1,4*^

^1^Key Laboratory of Plant Germplasm Enhancement and Specialty Agriculture, Wuhan Botanical Garden of the Chinese Academy of Sciences, Wuhan, 430074, P.R. China

^2^Department of Pharmacognosy and Pharmaceutical Botany, Faculty of Pharmaceutical Sciences, Chulalongkorn University, Bangkok 10330, Thailand

^3^Graduate University of Chinese Academy of Sciences, 19A Yuquanlu, Beijing, 100049, China

^4^Sino-African Joint Research Center, Chinese Academy of Sciences, Wuhan, 430074, China

^†^These authors contributed equally to this work.

*Corresponding author: Yuepeng Han; E-mail: yphan@wbgcas.cn

Postal address: Wuhan Botanical Garden of the Chinese Academy of Sciences, Wuhan, 430074, P.R. China

E-mail addresses:

Sornkanok Vimolmangkang: sornkanok.v@chula.ac.th

Albert Owiti: owitialbert@gmail.com

Collins Ogutu: collee52@gmail.com

Thitirat Meelaph: tammybio163@gmail.com

Xianbao Deng: dengxianbao@wbgcas.cn

Yuepeng Han: yphan@wbgcas.cn

**Legends of supplementary information**

Table S1. Primers for amplification of cDNA fragments of *NnNCS* genes in lotus.

Table S2. Primers for qRT-PCR analyses in lotus.

Figure S1. Standard curve of lotus alkaloid Nuciferine. Nuciferine dilution series was designed as follows: 10, 20, 40, 60, 80, 100, 150, 200 μg/mL in alkaloid extraction buffer (0.3 M HCl-methanol, 1:1 in volume).

Figure S2. Alignment of genomic DNA sequences of *NnNCS1* and *NnNCS2*. The intron sequences are highlighted in red color.

Figure S3. Alignment of the *NnNCS1* coding sequences and the putative cDNA sequences of *NnNCS6*.

Table S1. Primers for amplification of cDNA fragments of *NnNCS* genes in lotus

| Name | Forward (5’→3’) | Reverse (5’→3’) |
| --- | --- | --- |
| *NnNCS1* | ATGATGATCGGACGTGTAGTTAAC | TTAGTGGTTTCTCCTCTTTGATT |
| *NnNCS2* | AACGAAGAAAAAGATGATGATCGGC | TCAAGAATCATAAAATTCGGTGGACTTAG |
| *NnNCS3* | TATGCGTGGGCAAGTAACG | CTTAGACGTCACAAGCTTTAGCTTTC |
| *NnNCS4* | AATGCATGCTGGGCAACTATCA | CCTACTCTGTTTTAAGTTCAGTGAGATACTTC |
| *NnNCS5* | GGTGGAAGAAAATGATTCACAGTGTG | GGTTTCTCATTCATAGCATGCTTTAGC |
| *NnNCS6* | ACTTATGCCCGAAGTTTACAAGAG | TCACTTGTCGTCGAGCTCA |
| *NnNCS7* | ATGATGACTGCGCGTGTAAC | TCAAGAATCATAAAATTCCGCCAAT |

Table S2. Primers for qRT-PCR analyses in lotus

| Name | Forward (5’→3’) | Reverse (5’→3’) |
| --- | --- | --- |
| *NnNCS1* | GGTAGGCGTGCCTGCTGAC | ATCCCATCGGCGAGTTCG |
| *NnNCS2* | AGGTAGGCGTGCCTGCTGAT | GAGGGATAGCGCTCGCATATATAG |
| *NnNCS3* | CGAGGAGCGCTTAAAAGTGA | ATTAATCAGAGCCGCATTGG |
| *NnNCS4* | AGGAGGAGCCTTGGACCTT | GCTCCAGTGGCTTAGTGGTC |
| *NnNCS5* | GCTCGTCGTCAAATTGATGCCTC | CCATGTACGCGGCTCCATCATTT |
| *NnNCS6* | ATGGAACGGTCGGCACTGTCATTC | TCACTCGGACCAACTTCTCGCGT |
| *NnNCS7* | CTCTTCGTCCAACTTATGCCCGAA | CTTCCATATACGTGGCTTAGGAAGCG |
| *GADPH* | GGTGCTGAGTTCGTCGTAGA | TGGGAATGATGTTGAAGGAA |


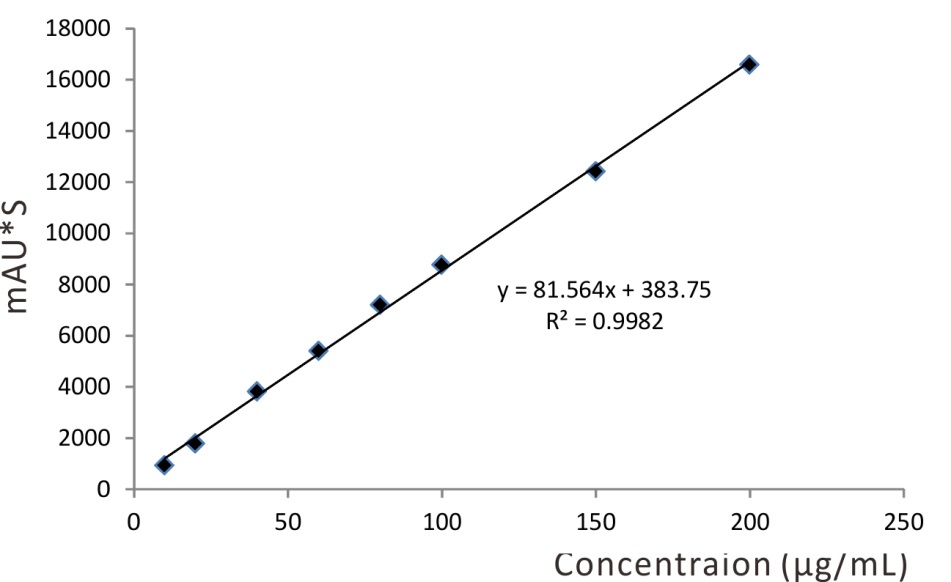


Figure S1. Standard curve of lotus alkaloid Nuciferine. Nuciferine dilution series was designed as follows: 10, 20, 40, 60, 80, 100, 150, 200 μg/mL in alkaloid extraction buffer (0.3 M HCl-methanol, 1:1 in volume).


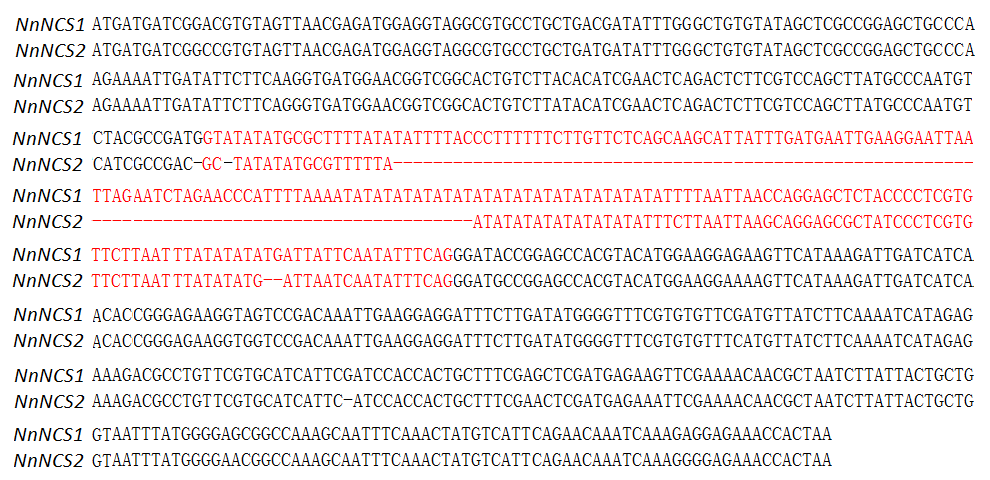


Figure S2. Alignment of genomic DNA sequences of *NnNCS1* and *NnNCS2*. The intron sequences are highlighted in red color.


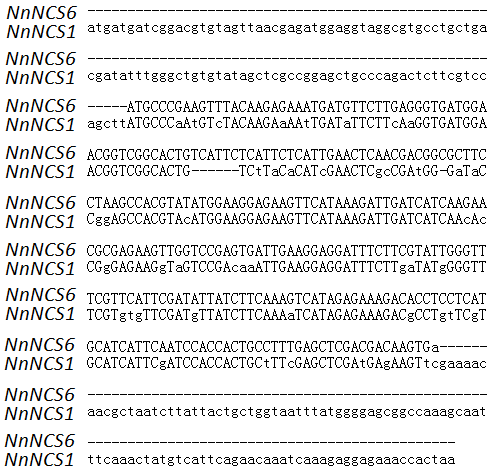


Figure S3. Alignment of the *NnNCS1* coding sequences and the putative cDNA sequences of *NnNCS6*.
